# Construction and evaluation of neonatal respiratory failure risk prediction model for neonatal respiratory distress syndrome

**DOI:** 10.1186/s12890-023-02819-4

**Published:** 2024-01-02

**Authors:** Yupeng Lei, Xia Qiu, Ruixi Zhou

**Affiliations:** 1 Department of Pediatrics, West China Second University Hospital, Sichuan University, Chengdu, 610041 Sichuan China; 2 Key Laboratory of Birth Defects and Related Diseases of Women and Children, Sichuan University, Ministry of Education, Chengdu, 610041 China

**Keywords:** NRDS, Respiratory failure, MIMIC-IV database, Nomogram, Predictive model

## Abstract

**Background:**

Neonatal respiratory distress syndrome (NRDS) is a common respiratory disease in preterm infants, often accompanied by respiratory failure. The aim of this study was to establish and validate a nomogram model for predicting the probability of respiratory failure in NRDS patients.

**Methods:**

Patients diagnosed with NRDS were extracted from the MIMIC-iv database. The patients were randomly assigned to a training and a validation cohort. Univariate and stepwise Cox regression analyses were used to determine the prognostic factors of NRDS. A nomogram containing these factors was established to predict the incidence of respiratory failure in NRDS patients. The area under the receiver operating characteristic curve (AUC), receiver operating characteristic curve (ROC), calibration curves and decision curve analysis were used to determine the effectiveness of this model.

**Results:**

The study included 2,705 patients with NRDS. Univariate and multivariate stepwise Cox regression analysis showed that the independent risk factors for respiratory failure in NRDS patients were gestational age, pH, partial pressure of oxygen (PO_2_), partial pressure of carbon dioxide (PCO_2_), hemoglobin, blood culture, infection, neonatal intracranial hemorrhage, Pulmonary surfactant (PS), parenteral nutrition and respiratory support. Then, the nomogram was constructed and verified.

**Conclusions:**

This study identified the independent risk factors of respiratory failure in NRDS patients and used them to construct and evaluate respiratory failure risk prediction model for NRDS. The present findings provide clinicians with the judgment of patients with respiratory failure in NRDS and help clinicians to identify and intervene in the early stage.

## Introduction

Neonatal respiratory distress syndrome (NRDS) is the most common respiratory system disease in premature babies, particularly those born before 28 weeks of gestation [1, 2]. It is caused by dysfunction of effective ventilation in neonates due to the lack of pulmonary surfactant (PS), or the immature development of the lung [3, 4]. Because of the formation of hyaline membrane in the pathophysiology of this disease, it is also called neonatal pulmonary hyaline membrane disease [5].The disease causes a progressive worsening of inspiratory dyspnea. NRDS patients may experience rapid breathing, grunting sounds while breathing, and flaring nostrils, they may also have a bluish tint to their skin due to inadequate oxygenation [6].NRDS has a high morbidity rate, 5% of near-term infants are affected, 30% of infants who had a gestational age of less than 30 weeks are affected, and 60% of premature infants who had a gestational age of less than 28 weeks are affected [7]. Many premature infants also die because of NRDS [8]. Severe NRDS can lead to neonatal respiratory failure(NRF), which is defined as decreased oxygen saturation and oxygen partial pressure (PO2), or the need for endotracheal intubation and mechanical ventilation [9]. NRF is likely to occur after NRDS for a period of time under the induction of various causes, affecting the development of children's circulatory system, nervous system, metabolism and other aspects, and even cause a serious impact on the prognosis of newborns [10].

At present, prenatal use of dexamethasone to promote fetal lung development and maturation [11], postpartum PS supplementation [12], and effective ventilation therapy [13] have reduced the incidence of NRDS, and also changed its severity and typical manifestations. However, NRDS remains the most common respiratory disease in preterm infants in the neonatal intensive care unit (NICU), and there are many cases of NRDS leading to NRF [14]. Therefore, being able to identify the cases with a high probability of developing NRF in NRDS patients is helpful for early medical intervention, and is of great significance for improving the prognosis of children.

Predictive models have been previously developed for neonatal respiratory distress syndrome in both preterm and late-preterm infants, as well as for predicting other complications associated with NRDS [15, 16]. Nevertheless, a predictive model for respiratory failure within the context of neonatal respiratory distress syndrome has yet to be established. A newborn refers to an infant who is in the initial 28 days of life after birth. during this neonatal period, infants diagnosed with NRDS are at a high risk of developing NRF. As such, this study aims to investigate the likelihood of NRF occurrence among neonates diagnosed with NRDS at both day 1 and day 28 after birth and then establishing a predictive model for the development of NRF in NRDS.

## Methods

### Data source

This study was a restrictive observational study from the Medical Information Mart for Intensive Care IV (MIMIC-IV version 1.0) database (https://physionet.org/content/mimiciv/1.0/), which is a large, freely accessible database of de-identified medical records for patients admitted to the intensive care unit (ICU) at the Beth Israel Deaconess Medical Center in Boston, Massachusetts, USA. It contains data from over 100,000 ICU stays between 2008 and 2019, making it one of the largest publicly available critical care datasets in the world [17]. The MIMIC-IV database includes information on patient demographics, vital signs, laboratory results, medications, diagnoses, procedures, and other clinical data. It also contains free-text nursing notes and physician progress notes, which can be used for natural language processing and other text-based analyses. The MIMIC-IV database has been used for a wide range of research studies, including machine learning and artificial intelligence approaches for predicting patient outcomes, developing clinical decision support systems, and improving patient care. It has also been used to investigate clinical questions related to sepsis, acute respiratory distress syndrome, cardiac arrest, and other critical care conditions. Individuals who have finished the Collaborative Institutional Training Initiative examination (Certification number 50366200 for YL) can access the database.

### Study population

In our study, we included neonatal patients with NRDS, and NRF secondary to the onset of NRDS. NRDS was determined following diagnostic codes from the International Classification of Diseases, 9th revised (ICD-9) and 10th revised (ICD-10) editions [18, 19] and we defined cases with a PaO2 level below 50 mmHg as neonatal respiratory failure [20, 21]. We extracted these patients’ parameters from the MIMIC-IV, and we collected the following data: basic information including gestational age, gender, ethnic group, admission time, onset time and discharge time. Then, biological variables were collected, including peripheral blood white blood cells (WBC), hemoglobin (Hb), platelets (PLT) from the blood routine examination; bilirubin from the blood biochemistry; pH, PO2, partial pressure of carbon dioxide (PCO2) from the blood gas analysis; blood culture and cerebrospinal fluid (CSF) culture results. All data were collected within 48 h of patient admission, and in cases with multiple measurements, we analyzed only the initial measurements. The clinical variables mainly included intrauterine growth retardation (IUGR), neonatal asphyxia, neonatal apnea, neonatal jaundice, neonatal intracranial hemorrhage, neonatal coagulation disorders, neonatal pneumonia, neonatal anemia and infection. Treatment measures included whether or not to use PS, whether or not to use noninvasive ventilation, whether or not to use caffeine, and whether or not to use parenteral nutrition. The code of data extraction is available on Github (https://github.com/MIT-LCP/mimic-iv).

### Statistical analysis

For nomogram construction and validation, we randomly divided all the NRDS patients into training and validation cohorts, in a ratio of 7:3 [22]. The demographic and clinical characteristics of the patients were described in the training and validation datasets. Univariate Cox and stepwise Cox regression analysis were used to screen variables. *P* values of less than 0.05 (*P* < 0.05) in univariate Cox regression analysis were included in the multivariate Cox proportional hazards regression analysis. To simplify the model and prevent collinearity of variables, multivariate Cox proportional hazards regression analysis was performed to identify variables that significantly affected the onset of NRF, using a significance threshold (*P* < 0.05) [23]. These eligible variables were included in the final Cox proportional hazards model, and the corresponding nomogram was drawn. The predicted values of the nomogram were calculated, and the actual values observed were compared with the results of the nomogram. The calibration curve [24], receiver operating characteristic (ROC) curve [25] and decision curve [26] were drawn to test the performance of the model. All statistical analyses were conducted using R 4.2.1 (https://www.r-project.org/). In the R software package used, TableOne (0.13.2) was used for data description, survival (3.2.13) was used for feature selection, and RMS (6.2.0) was used for model construction and nomogram drawing. Bilateral *P* < 0.05 was considered to indicate statistical significance.

## Results

### Patient characteristics

A total of 2705 patients diagnosed with NRDS between 2008 and 2019 were included in this study, and NRF was observed in 1194 (44.1%) of them. The training and validation cohorts of NRDS patients consisted of 1899 and 806 cases, respectively. In the total cohort of NRDS patients, the majority of patients were white (30.7%) and male (57.6%). Patients with infection accounted for 16.1% and 17.5% of those in the training and validation cohorts, respectively, while patients with IUGR accounted for 8.1% and 7.0%, and those with neonatal asphyxia accounted for 0.4% and 0.1%. From the laboratory test results, the median pH in both cohorts were 7.29 [7.24, 7.34]. The median WBC in the training and validation cohorts were respectively 10.30 [7.00, 15.20] and 10.40 [6.90, 14.40]. The median PO2 in both cohorts were 47.00 [39.00, 58.00]. Patients with positive blood culture accounted for 5.7% and 4.8% of those in the training and validation cohorts, respectively, and neonatal respiratory failure patients accounted for 44.1% and 44.2%. The remaining baseline characteristics are listed in Table 1. And there was no significant statistical difference between these variables in the training and validation cohorts (*P* > 0.05).

**Table 1:** Characteristics in the study about patients with NRDS

Characteristics	Total cohort	Training cohort	Validation cohort	*p*
Population	2705	1899	806	
NRF population	1194 (44.1%)	838 (44.1%)	356 (44.2%)	
Gender (Male) (%)	1558 (57.6)	1098 (57.8)	460 (57.1)	0.751
Gestational age < 28 weeks (%)	382 (26.8)	267(27.1)	115 (26.1)	0.433
Ethnicity (%)				0.12
Asian	184 (6.8)	136 (7.2)	48 (6.0)	
Black	312 (11.5)	231 (12.2)	81 (10.0)	
White	831 (30.7)	581 (30.6)	250 (31.0)	
Other	1378 (50.9)	951 (50.1)	427 (53.0)	
pH (median [IQR])	7.29 [7.23, 7.34]	7.29 [7.24, 7.34]	7.29 [7.23, 7.34]	0.537
PCO_2_ (mmHg) (median [IQR])	51.00[44.00, 60.00]	51.00 [44.00, 60.00]	51.00 [43.00, 60.00]	0.991
PO_2_ (mmHg) (median [IQR])	47.00[39.00, 58.00]	47.00 [39.00, 58.00]	47.00 [39.00, 58.00]	0.895
WBC (× 10^9^ /L) (median [IQR])	10.30 [6.97, 14.93]	10.30[7.00,15.20]	10.40[6.90,14.40]	0.471
Hb (g/dL) (median [IQR])	15.80 [14.40, 17.30]	15.90 [14.40, 17.30]	15.80 [14.40, 17.38]	0.961
PLT (× 10^9^ /L) (median [IQR])	248.00 [201.00, 299.00]	247.00 [200.00, 300.00]	250.00 [202.00, 297.00]	0.417
Bilirubin (mg/dL) (median [IQR])	5.50 [4.10, 7.80]	5.50 [4.10, 7.75]	5.50 [4.00, 8.10]	0.602
Blood culture (%)				0.412
Not examined	130 (4.8)	103 (5.4)	27 (3.3)	
Negative	2428 (89.8)	1688 (88.9)	740 (91.8)	
Positive	147 (5.4)	108 (5.7)	39 (4.8)	
CSF culture (%)				0.468
Not examined	2399 (88.7)	1687 (88.8)	712 (88.3)	
Negative	295 (10.9)	206 (10.8)	89 (11.0)	
Positive	11 (0.4)	6 (0.3)	5 (0.6)	
Disease time (median [IQR])	3.96 [0.13, 22.39]	3.98 [0.13, 22.73]	3.96 [0.13, 21.31]	0.576
Infection (%)	446 (16.5)	305 (16.1)	141 (17.5)	0.389
IUGR (%)	214 (7.8)	156 (8.1)	58 (7.0)	0.378
Neonatal anemia (%)	818 (29.8)	579 (30.1)	239 (29.0)	0.599
Neonatal apnea (%)	1735 (63.1)	1204 (62.6)	531 (64.4)	0.376
Neonatal asphyxia (%)	8 (0.3)	7 (0.4)	1 (0.1)	0.487
Neonatal coagulation disorders (%)	119 (4.3)	87 (4.5)	32 (3.9)	0.515
Neonatal intracranial Hemorrhage (%)	119 (4.3)	87 (4.5)	32 (3.9)	0.515
Neonatal pneumonia (%)	43 (1.6)	31 (1.6)	12 (1.5)	0.895
Caffeine (%)	1185 (43.1)	827 (43.0)	358 (43.4)	0.855
PS (%)	1006 (36.6)	705 (36.6)	301 (36.5)	0.989
Parenteral nutrition (%)	1339 (48.7)	932 (48.4)	407 (49.4)	0.677
Respiratory support (%)	1850 (68.4)	1313 (69.1)	537 (66.6)	0.214

*IQR* Interquartile range, *NRF* Neonatal respiratory failure, *PCO*_*2*_ Partial pressure of carbon dioxide, *PO*_*2*_ Partial pressure of oxygen, *WBC* White blood cells, *Hb* Hemoglobin, *PLT* Platelets, *CSF* Cerebrospinal fluid, *IUGR* Intrauterine growth retardation, *PS* Pulmonary surfactant

#### Screening for pathogenic factors of neonatal respiratory failure.

For NRDS patients, based on univariate and stepwise Cox regression analysis, we identified 11 independent prognostic factors in the training cohort. Gestational age < 28 weeks (hazard ratio (HR) = 6.63(5.59–7.85), *P* < 0.0001), pH (HR = 0.05 (0.02–0.13), *P* < 0.0001), PO2 (HR = 0.96 (0.95–0.96), *P* < 0.0001), PCO2 (HR = 0.99 (0.98–1), *P* < 0.05), Hb (HR = 0.91(0.88–0.93), *P* < 0.0001), Blood culture (HR = 3.85(1.88–7.89), *P* < 0.0001), infection (HR = 1.34(1.11–1.61), *P* < 0.05), Neonatal intracranial Hemorrhage (HR = 1.42(1.08–1.86), P < 0.05), PS (HR = 0.76(0.65–0.89), *P* < 0.0001), parenteral nutrition (HR = 2.13(1.78–2.54), *P* < 0.0001) and noninvasive ventilation (HR = 0.64(0.55–0.73), *P* < 0.0001), were all significantly associated with neonatal respiratory failure in NRDS patients (Table 2).

**Table 2:** Univariate and multivariate Cox regression analysis based on all variables for neonatal respiratory failure in NRDS patients

Characteristics	Univariate analysis		Multivariate analysis	
	HR (95%CI)	*P* value	HR (95%CI)	*P* value
Ref: Ethnicity (Asian)				
Ethnicity (White)	1.23 (0.93–1.62)	0.1391		
Ethnicity (Black)	0.96 (0.69–1.33)	0.8157		
Ethnicity (Other)	0.89 (0.66–1.19)	0.4157		
Gender (Male)	0.95(0.83–1.09)	0.4405		
Gestational age < 28 weeks (%)	6.21(5.37–7.19)	< 0.0001	6.63(5.59–7.85)	< 0.0001
pH	0.12 (0.06–0.24)	< 0.0001	0.05 (0.02–0.13)	< 0.0001
PCO_2_	1.01 (1.01–1.02)	< 0.0001	0.99 (0.98–1)	0.0032
PO_2_	0.98 (0.98–0.99)	< 0.0001	0.96 (0.95–0.96)	< 0.0001
WBC	0.96 (0.94–0.97)	< 0.0001		
Hb	0.94(0.91–0.96)	< 0.0001	0.91(0.88–0.93)	< 0.0001
PLT	1(1–1)	0.9204		
Bilirubin	1(1–1)	< 0.0001		
Ref: Blood culture (not examined)				
Blood culture (negative)	6.63(3.44–12.79)	< 0.0001	3.61(1.86–7)	< 0.0001
Blood culture (positive)	10.12(5.04–20.31)	< 0.0001	3.85(1.88–7.89)	< 0.0001
Ref: CSF culture (not examined)				
CSF culture (negative)	2(1.67–2.41)	< 0.0001		
CSF culture (positive)	1.58(0.51–4.91)	0.4305		
Infection	2.2(1.88–2.57)	< 0.0001	1.34(1.11–1.61)	0.002
IUGR	1.12(0.87–1.44)	0.369		
Neonatal anemia	1.26(1.09–1.45)	0.0017	1.16(0.97–1.39)	0.1018
Neonatal apnea	2.8(1.26–6.26)	0.0119		
Neonatal asphyxia	2.31 (1.04–5.16)	0.0409		
Neonatal coagulation disorders	2.86 (2.26–3.63)	< 0.0001		
Neonatal intracranial Hemorrhage	3.1(2.43–3.94)	< 0.0001	1.42(1.08–1.86)	0.0107
Neonatal pneumonia	1.44(0.9–2.3)	0.1242		
Caffeine	1.28(1.12–1.47)	< 0.0001	0.87(0.73–1.04)	0.1377
PS	0.77(0.67–0.89)	< 0.0001	0.76(0.65–0.89)	< 0.0001
Parenteral nutrition	2.24(1.95–2.58)	< 0.0001	2.13(1.78–2.54)	< 0.0001
Respiratory support	0.59(0.51–0.67)	< 0.0001	0.64(0.55–0.73)	< 0.0001

*PCO*_*2*_ Partial pressure of carbon dioxide, *PO*_*2*_ Partial pressure of oxygen, *WBC* White blood cells, *Hb* Hemoglobin, *HR* Hazard ratio, *PLT* Platelets, *CSF* Cerebrospinal fluid, *IUGR* Intrauterine growth retardation, *PS* Pulmonary surfactant

#### Nomogram construction

**Fig. 1:**
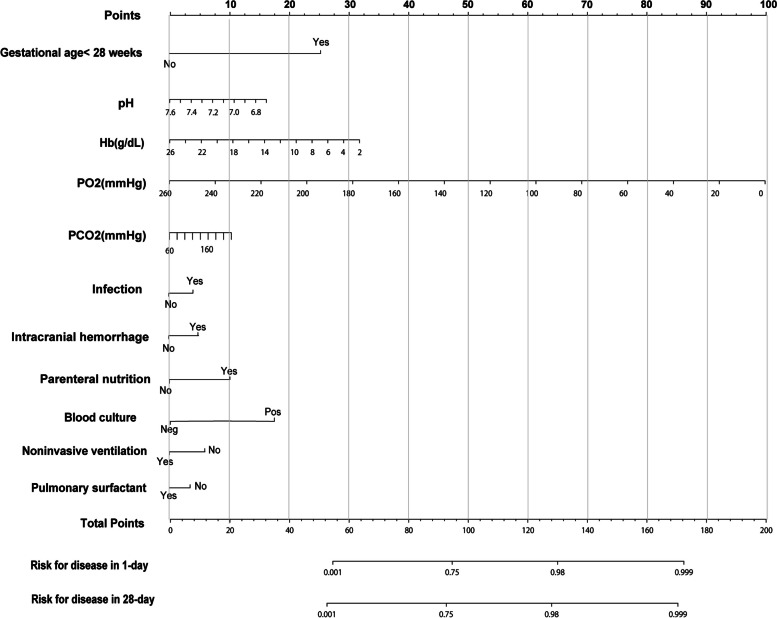
This nomogram estimates the likelihood of neonatal respiratory failure (NRF) in patients diagnosed with neonatal respiratory distress syndrome (NRDS). When using the nomogram, draw a vertical line from each variable to the points scale, noting the corresponding score, and then sum the scores for all variables to get a total. Finally, refer to the bottom of the nomogram to determine the predicted probability of NRF based on the total score. For comorbidities, 'Yes' indicates the presence and 'No' indicates the absence of the condition. For laboratory test results, 'Neg' stands for negative, and 'Pos' for positive. For treatment measures, 'Yes' indicates the measure was applied, while 'No' means it wasn't. PCO_2_, partial pressure of carbon dioxide; PO_2_, partial pressure of oxygen; Hb, hemoglobin

We developed a nomogram predicting the occurrence of NRF at day 1 and day 28 in patients with NRDS, based on the selected pathogenic factors from the training cohort (Fig. 1). Each level of every variable was assigned a score based on the points scale. The total score was obtained by adding the scores of each of the selected variables. The prediction corresponding to this total score then helped in estimating the occurrence of NRF within day 1 and day 28 for each NRDS patients.

#### Nomogram validation

**Fig. 2:**
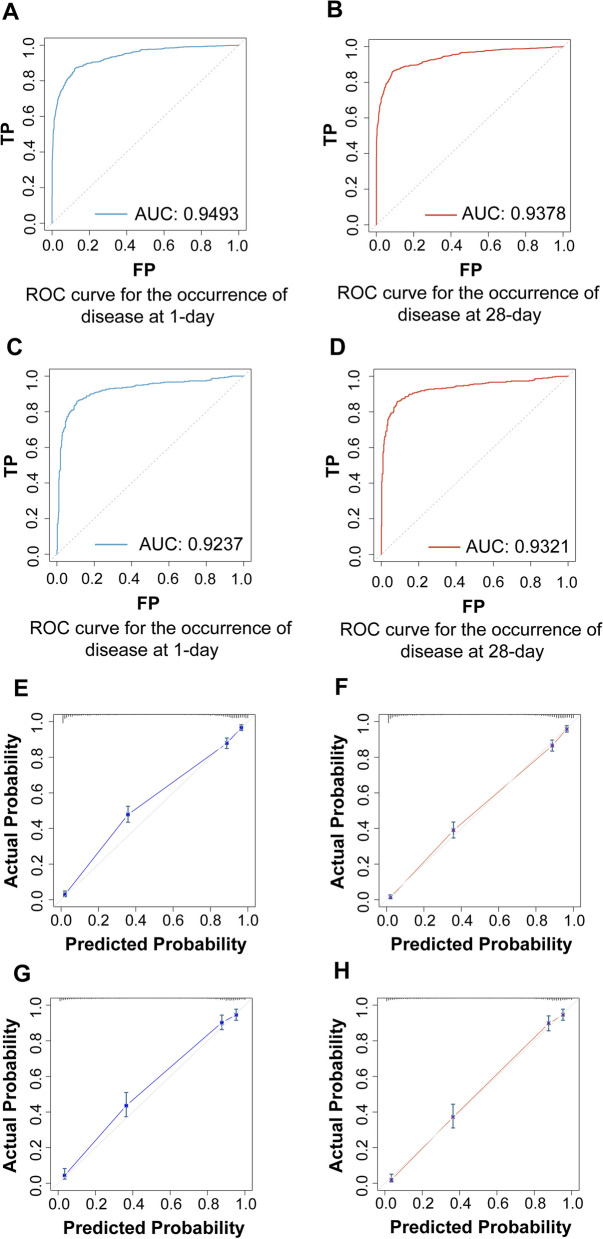
ROC and calibration curves for both the training and validation cohorts. **A** ROC curve representing disease occurrence on day 1 for the training cohort; **B** ROC curve for disease occurrence on day 28 in the training cohort; **C** ROC curve for disease occurrence on day 1 in the validation cohort; **D** ROC curve for disease occurrence on day 28 in the validation cohort. **E** Calibration curve for disease occurrence on day 1 in the training cohort; **F** Calibration curve for disease occurrence on day 28 in the training cohort; **G** Calibration curve for disease occurrence on day 1 in the validation cohort; **H** Calibration curve for disease occurrence on day 28 in the validation cohort. ROC, receiver operating characteristic; AUC, the area under the ROC curve. TP, true positive; FP, False Positive

**Fig. 3:**
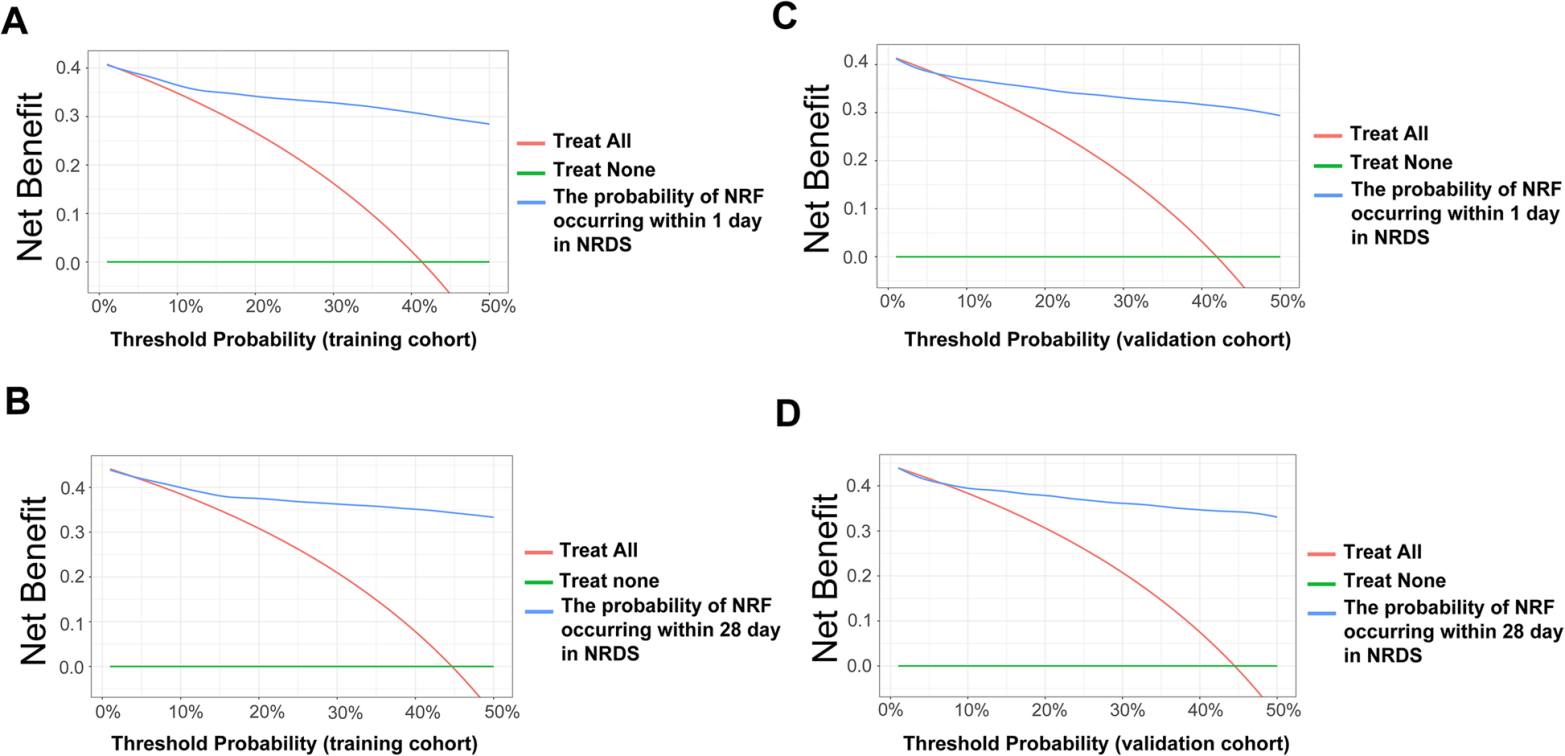
Decision-curve analysis of the validation cohort and the training cohort. **A** The occurrence of disease at day 1 in the training cohort; **B** the occurrence of disease at day 28 in the training cohort; **C** the occurrence of disease at day 1 in the validation cohort; (**D**) the occurrence of disease at day 28 in the validation cohort

We detected the ability to predict NRF in NRDS patients from the nomogram. Figure 2 indicates that the area under the ROC curve (AUC) values of the nomogram were 0.9343 (Fig. 2A) and 0.9378 (Fig. 2B) for the occurrence of disease within day 1- and day 28- in the training cohort, respectively, and in the validation cohort, the AUC values of the nomogram were 0.9237 (Fig. 2C) and 0.9321 (Fig. 2D). It shows that our model has good predictive ability in both the training and validation cohorts [27]. Figure 2 also displays the calibration curves of the nomogram. The calibration curves of the training (Fig. 2E/F) and validation (Fig. 2G/H) cohorts indicate that the nomogram provided a good fit to the data, and that our models did not significantly overestimate or underestimate risk [28]. Finally, we drew a decision curve analysis to illustrate the clinical applicability of the nomogram (Fig. 3). It indicated that clinical interventions guided by our nomogram had a high net benefit [26].

## Discussion

NRF secondary to NRDS is not uncommon, it may occur after NRDS for a period of time after the onset of NRDS, especially when combined with multiple risk factors. We performed a large sample multi-risk factor analysis, and indicated Gestational age < 28 weeks, pH, PO2, PCO2, Hb, Blood culture, infection, Neonatal intracranial Hemorrhage, PS, parenteral nutrition and respiratory support as independent risk factors for NRF in NRDS patients. These results were used to construct a nomogram for estimating the NRF risk in NRDS patients within day 1 and day 28 during hospitalization. The validity of our nomogram model was determined using multiple indicators, including AUC, calibration curves and decision-curve analysis. In this study, we constructed a more comprehensive model based on a combination of various risk factors, to better predict the risk of NRF in patients with NRDS.

We found that most of the secondary NRF in NRDS patients occurred within one day [29]. This is also consistent with the clinical features of NRDS, which is a progressive worsening of dyspnea that develops gradually after birth, therefore most NRDS patients typically develop respiratory failure within 1 day. Premature infants with a gestational age of less than 28 weeks are at an increased risk of developing NRF following NRDS. This is primarily due to the fact that premature infants exhibit underdeveloped lungs, insufficient production of surface-active substances, and compromised immunity, which collectively increase the likelihood of disease progression and exacerbation2. In addition, we found that infection-related factors were also closely related to neonatal respiratory failure secondary to NRDS, including clear presence of infection-related symptoms, or positive microbial tests such as blood culture and CSF culture, which may be due to the decreased activity and increased degradation of PS caused by inflammatory mediators [30]. At the same time, inflammation can cause mechanical damage to type II alveolar epithelial cells, and further reduce the secretion of PS [31]. Thus, patients with pathogen cultures detected during the first time should receive clinical attention. Antimicrobial agents should include all possibly present pathogenic bacteria in the initial stage of anti-infective therapy.

In terms of treatment, parenteral nutrition increases the risk of NRF, which may be associated with infection due to parenteral nutrition, or increased pulmonary circulation due to excessive fluid intake [32]. Therefore, rational parenteral nutrition and fluid management are critical in patients with NRDS. At the same time, the use of noninvasive ventilation and Surfactant replacement can effectively reduce the occurrence of NRF. Noninvasive ventilation techniques, like nasal Continuous Positive Airway Pressure (nCPAP), offer positive end-expiratory pressure to NRDS patients. This aids in consistently expanding the alveoli, enhancing gas exchange, and subsequently mitigating the risk of NRF. As the respiratory distress in NRDS patients stems from a PS deficiency, replenishing PS further reduces the likelihood of NRF [33].

Blood gas analysis is an important laboratory test index in neonatal respiratory management. Our study found that pH, PO2 and PCO2 are of great importance to NRF [10]. These indicators can not only reflect the occurrence of NRF, but also be used as risk factors to early judge NRF secondary to NRDS, and remind us to carry out early intervention. Our findings revealed a significant association between reduced hemoglobin levels and disease development, potentially attributed to inadequate oxygenation among anemic children. Furthermore, the impact of intracranial hemorrhage on disease onset may be related to the central nervous system's role in respiratory regulation.

Clinical predictive models can be used to study the relationship between future outcome events and baseline status in patients [34]. They can integrate the results of traditional analyses, simplify them with more intuitive and convincing presentations, and predict the probability of certain outcome events with a scoring system [35]. NRDS is the most common respiratory disease in preterm infants. NRF caused by NRDS can be followed by multiple organ dysfunctions, which has a great impact on the prognosis of preterm infants. At present, the risk factors of respiratory failure secondary to NRDS have not been well studied. Therefore, the establishment of this prediction model has important clinical significance for early identification of NRF in patients with NRDS. Our doctors can use the scoring results of the model to communicate with the family members of the neonate, help them understand the severity of the child's condition, work out a treatment plan together, improve the degree of cooperation, and prevent the occurrence of NRF to the greatest extent. However, the predictive ability of this nomogram may be improved by considering other potential important factors that we were not able to obtain from the MIMIC-IV database, such as maternal factors during pregnancy, perinatal medication and detailed insights into the parameters associated with non-invasive ventilation. And although the number of patients included was large, this study is a single-center study, and lacks external validation.

## Conclusion

This study identified the independent risk factors of respiratory failure in NRDS patients and used them to construct and evaluate respiratory failure risk prediction model for NRDS. The present findings provide clinicians with the judgment of patients with respiratory failure in NRDS and help clinicians to identify and intervene in the early stage.

## Data Availability

The datasets analyzed during the current study are available in the MIMIC-IV repository, https://physionet.org/content/mimiciv/1.0/.

## Abbreviations

AUC: Area under the ROC curve
CSF: Cerebrospinal fluid
Hb: Hemoglobin
IUGR: Intrauterine growth retardation
ICD: International Classification of Diseases
MIMIC: Medical Information Mart for Intensive Care
nCPAP: Nasal Continuous Positive Airway Pressure
NICU: Neonatal intensive care unit
NRDS: Neonatal respiratory distress syndrome
NRF: Neonatal respiratory failure
PCO2: Partial pressure of carbon dioxide
PLT: Platelets
PO2: Partial pressure of oxygen
PS: Pulmonary surfactant
ROC: Receiver operating characteristic curve
WBC: White blood cells
